# Tumor suppressor role of cytoplasmic polyadenylation element binding protein 2 (CPEB2) in human mammary epithelial cells

**DOI:** 10.1186/s12885-019-5771-5

**Published:** 2019-06-11

**Authors:** Joshua Tordjman, Mousumi Majumder, Mehdi Amiri, Asma Hasan, David Hess, Peeyush K. Lala

**Affiliations:** 1 Department of Anatomy and Cell Biology, University of Western Ontario, London, Ontario N6A5C1 Canada; 2 Department of Biology, Brandon University, Brandon, Manitoba R7A6A9 Canada; 3 Department of Physiology and Pharmacology, University of Western Ontario, London, Ontario N6A5C1 Canada; 4 Department of Oncology, University of Western Ontario, London, Ontario N6A5C1 Canada

**Keywords:** CPEB2, Tumor suppressor, COX-2, EMT, EP4 receptor, Breast Cancer, Stem-like cells, MicroRNA-526b, MicroRNA-655, p53, Polysome profiling

## Abstract

**Background:**

Over-expression of cyclooxygenase (COX)-2 promotes breast cancer progression by multiple mechanisms, including induction of stem-like cells (SLC). Combined gene expression and microRNA microarray analyses of empty vector vs *COX-2-* transfected COX-2 low MCF7 breast cancer cell line identified two COX-2-upregulated microRNAs, miR-526b and miR-655, both found to be oncogenic and SLC-promoting. Cytoplasmic Polyadenylation Element-Binding Protein 2 (CPEB2) was the single common target of both microRNAs, the functions of which remain controversial. CPEB2 has multiple isoforms (A-F), and paradoxically, a high B/A ratio was reported to impart anoikis-resistance and metastatic phenotype in triple- negative breast cancer cells. We tested whether CPEB2 is a tumor suppressor in mammary epithelial cells.

**Methods:**

We knocked-out *CPEB2* in the non-tumorigenic mammary epithelial cell line MCF10A by CRISPR/Cas9-double nickase approach, and knocked-down *CPEB2* with siRNAs in the poorly malignant MCF7 cell line, both lines being high *CPEB2*-expressing. The resultant phenotypes for oncogenity were tested in vitro for both lines and in vivo for *CPEB2KO* cells. Finally*, CPEB2* expression was compared between human breast cancer and non-tumor breast tissues.

**Results:**

*CPEB2* (isoform A) expression was inversely correlated with *COX-2* or the above microRNAs in *COX-2*-divergent breast cancer cell lines. CPEB2KO MCF10A cells exhibited oncogenic properties including increased proliferation, migration, invasion, EMT (decreased E-Cadherin, increased Vimentin, N-Cadherin, SNAI1, and ZEB1) and SLC phenotype (increased tumorsphere formation and SLC marker-expression). Tumor-suppressor p53 protein was shown to be a novel translationally-regulated target of CPEB2, validated with polysome profiling. CPEB2KO, but not wild-type cells produced lung colonies upon intravenous injection and subcutaneous tumors and spontaneous lung metastases upon implantation at mammary sites in NOD/SCID/IL2Rϒ-null mice, identified with HLA immunostaining. Similarly, siRNA-mediated *CPEB2* knockdown in MCF7 cells promoted oncogenic properties in vitro*.* Human breast cancer tissues (*n* = 105) revealed a lower mRNA expression for *CPEB2* isoform A and also a lower A/B isoform ratio than in non-tumour breast tissues (*n* = 20), suggesting that CPEB2A accounts for the tumor-suppressor functions of CPEB2.

**Conclusions:**

CPEB2, presumably the isoform A, plays a key role in suppressing tumorigenesis in mammary epithelial cells by repressing EMT, migration, invasion, proliferation and SLC phenotype, via multiple targets, including a newly-identified translational target p53.

**Electronic supplementary material:**

The online version of this article (10.1186/s12885-019-5771-5) contains supplementary material, which is available to authorized users.

## Background

Upregulation of cyclooxygenase (COX)-2, an inflammation-associated enzyme, noted in half of the breast cancer patients [1] promotes tumor progression and metastasis through multiple mechanisms, including increased cancer cell proliferation, migration, invasion, epithelial-to-mesenchymal transition (EMT), tumor-associated angiogenesis, lymphangiogenesis, and induction of stem-like cells (SLCs) [2–6]. SLCs represent a dynamic state regulated by the tumor micro-environment [7] and resist chemo- and radiation therapies, causing tumor recurrence [8, 9]. COX-2 mediated stimulation of various oncogenic events in breast cancer, as listed above, is largely due to the activation of the PGE receptor EP4 by the endogenous Prostaglandin (PG)E2 [10].

Combined gene and microRNA expression microarrays with ectopic *COX-2* overexpressing and Mock (empty vector-transfected) MCF7 breast cancer cells [6] revealed 26 genes that are downregulated, along with two microRNAs, miR-526b [11] and miR-655 [12] that are upregulated by *COX-2*. MicroRNAs silence their gene targets either by degrading the mRNAs or blocking their translation [13]. We found that both miRNAs - miR-526b and miR-655 were oncogenic and SLC-promoting [11, 12]. The only COX-2 down-regulated gene targeted by both microRNAs was identified as Cytoplasmic Polyadenylation Element-Binding Protein 2 (*CPEB2*), the functions of which in tumor biology remain controversial.

The CPEB family includes 4 members (CPEB1–4) which regulate translation of their target mRNAs by binding to a Cytoplasmic Polyadenylation Element (CPE) in the 3’untranslated region [14]. Polyadenylation in their target mRNAs depends on both a CPE sequence (UUUUUAAU) and a polyadenylation hexanucleotide signal (AAUAAA) [15]. CPEB proteins can repress or activate translation of target mRNAs by respectively shortening or elongating the poly-A tail [16]. CPEB1 was shown to be a tumor-suppressor, depletion of which in mammary epithelial cells led to Epithelial-Mesenchymal-Transition (EMT) and metastatic phenotype [17]. It restrained proliferation of glioblastoma cells by activating p27 mRNA reanslation [18]. CPEB3 appeared to be a tumor-suppressor, targeted by oncogenic miR-107 in hepatocellular carcinoma [19]. Furthermore, a high CPEB3 protein expression was associated with increased survival in renal cell carcinoma patients [20]. The roles of CPEB4 in tumors remain conflicting. A tumor- suppressor role was demonstrated by its being the target of an oncogenic miR-550A in hepatocellular carcinoma [21]. However, CPEB4 mediated translational activation of oncogenic mRNAs in pancreatic cancer [22], and EMT induction, growth and metastasis in gastric cancer cells [23] illustrate pro-oncogenic functions.

The role of CPEB2 in cancer remains paradoxical. A tumor-suppressor role was suggested by CPEB2 binding to HIF1α mRNA and suppressing its translation under normoxic conditions, but releasing it to allow translation under hypoxic conditions [24]. This results from interaction with the elongation factor eEF2 [25]. HIF1α is short-lived under normoxic conditions, but stabilized under hypoxic conditions to stimulate genes promoting angiogenesis, EMT, migration, SLC functions, metastasis and therapeutic resistance [26]. Binding of CPEB2 to the mesenchymal transcription factor TWIST1 down-regulated its translation [27], suggesting an EMT-suppressor function. A tumor-suppressor role of CPEB2 was further suggested by its down-regulation by microRNA-885-5p, a mediator of EMT, tumorigenesis and metastasis in colorectal cancer [28].

The roles of CPEB2 in breast cancer appear to be complex, depending on the expression of different CPEB2 isoforms. CPEB2 has six isoforms (A-F). By selecting cells for anoikis-resistance in vitro from triple-negative breast cancer (TNBC) cell lines, Johnson et al. [29] reported that an alternative splicing of *CPEB2*, leading to a high isoform B:A ratio mediated anoikis-resistance and metastatic phenotype. They suggested that isoform A which excludes exon 4 is a tumor- suppressor, whereas isoform B that includes exon 4, is a tumor-promoter. This suggestion was validated by next generation sequencing and isoform-specific down-regulation of CPEB2A and B in TNBC lines [30]. They concluded that CPEB2B plays an antagonistic role against CPEB2A by alleviating the translational inhibition of HIF-1α and Twist 1 imparted by CPEB2A.

We adopted a different approach to examine the functions of CPEB2 in breast epithelial cell lines, by depleting the entire *CPEB2* gene and observing the resultant phenotypic changes: (a) *CPEB2* was knocked out using a double nickase CRISPR plasmid in an immortalized non-tumorigenic human mammary epithelial cell line MCF10A, reported to be a reliable model for normal mammary epithelium [31]; (b) *CPEB2* was knocked down with siRNAs in the MCF7 cell line, a mammary carcinoma of low malignancy [6]. *CPEB2*KO MCF10A cells exhibited an oncogenic phenotype in vitro, as indicated by increased proliferation, migration, invasion, EMT, stimulation of SLC and a downregulation of p53 tumor suppressor protein owing to a decreased translation of *p53* mRNA. They exhibited lung colonization after intravenous injection and subcutaneous tumorigenicity upon inoculation at the mammary sites in NOD/SCID/IL2Rγ null mice. SiRNA-mediated *CPEB2* knockdown in MCF7 breast cancer cells also resulted in enhanced oncogenic properties tested in vitro*.* These results confirm *CPEB2* as a tumor-suppressor in breast epithelial and poorly malignant breast cancer cells likely resulting from the CPEB2A isoform prevalence in these cells. This contention was supported by a higher CPEB2A isoform expression and A: B isoform ratio in non-tumorous human breast tissues than in cancerous breast tissues.

## Materials and methods

### Cell culture

The immortalized non-tumorigenic mammary epithelial cell line MCF10A (ATCC, at 4–6 passages) was cultured in DMEM (Gibco, Thermofisher, CA) supplemented with 5% horse serum (Invitrogen, Thermofisher, CA), 20 ng/ml EGF, 0.5 mg/ml hydrocortisone (Sigma, Oakville, ON, CA), 100 ng/ml cholera toxin (Sigma), 10 μg/ml insulin (Sigma), and 1% penicillin/streptomycin (Invitrogen) [31]. MCF7 cells (ATCC, Manassas, VA, USA, at 4–6 passages) were grown in EMEM supplemented with 10% FBS, 100 μg/mL of penicillin/streptomycin (Gibco) and 10 μg/mL of insulin (Sigma).

### CRISPR knockout of CPEB2 and SiRNA mediated CPEB2 knockdown

Total *CPEB2* was knocked out in MCF10A cells with a CRISPR double nickase plasmid (Santa Cruz, Dallas, TX, USA) targeting exon 1 of the gene, that combines a Cas9 nickase mutant with pairs of guide RNAs to introduce targeted double-strand breaks, ensuring a high knockout specificity [32]. Cells transfected with Amaxa Cell Line Nucleofector Kit IV (Lonza, Allendale, NJ, USA) were subjected to 72 h of puromycin selection and expanded. MCF7 cells were transfected with 1 μM of either *CPEB2* siRNA (a pool of siRNAs that gave the best results) or Universal Scrambled Control siRNA (OriGene, Rockville, MD, USA). After 24 h media was changed and experiments conducted at 48 h. CPEB2 downregulation in KO and KD cells was validated with qRT-PCR.

### Protein extraction and Western blot

Proteins extracted from cell lysates were subjected to western immunoblots [6], using primary antibodies at the following dilutions: CPEB2 (Origene cat # TA344026, rabbit polyclonal, 1:1000; lacking isoform-specificity), E-Cadherin (Cell Signalling, Danvers, Mass, USA, rabbit monoclonal, 1:1000), Vimentin (Cell Signalling, rabbit monoclonal, 1:1000)*,* β-actin (Santa Cruz, mouse monoclonal, 1:4000 or Cell Signalling, rabbit monoclonal, 1:1000), N-Cadherin (Santa Cruz, Rabbit polyclonal, 1:400), p53 and p21 (both from Novus Biologicals, Centennial, Colorado, USA, mouse monoclonal, 1:200) and β-Catenin (Sigma, rabbit polyclonal, 1:4000). After primary antibody incubation overnight, membranes were incubated for 1 h with appropriate secondary antibody at the following dilutions: Goat Anti-Rabbit (1:10000, Li-COR, Lincoln, Nebraska, USA) and Donkey Anti-Mouse (1:10000, Li-COR). Membranes were scanned using the Odyssey Infrared Imaging System (Li-COR).

### Immunofluorescence

#### Monolayers

Cells grown on glass coverslips were treated with various primary antibodies as reported [6]: E-Cadherin (Cell Signalling, 1:500), Vimentin (Cell Signalling, 1:500), and N-Cadherin (Sigma, 1:500). The cells were then incubated with fluorochrome-conjugated secondary antibodies (Biotum, Cedarlane, Burlington, ON, CA) at the following dilutions: Goat Anti-Rabbit 594 (1:500), Goat Anti-Rabbit 488 (1:500) and Goat Anti-Mouse 488 (1:500). Vectashield anti-fade mounting medium with DAPI (Vector-labs, Burlignton, ON, CA) was used to mount the slides. Fluorescent images were taken with Zeiss LSM 510 Meta Multiphoton Confocal Microscope, and fluorescence intensities calculated with ImageJ software. The raw integrated density was calculated for each cell and normalized to the cell area. Data were presented as an average of all cells.

#### Tumorspheres (spheroids)

Fixation, permeabilization, and antibody staining for tumorspheres (minimum 60 μm diameter) were conducted as reported [6]. Incidence of fluorescent cells (for ALDH1, NANOG, and SOX-2) was computed among total number of cells marked by DAPI.

### Migration and invasion assays using Transwells

Semi-confluent MCF10A and MCF7 cells were serum-starved overnight and seeded on microporous membranes (8 μm pore diameter) coated without or with Matrigel respectively for migration and invasion assays using transwell inserts [6]. Bottom chambers included 5% horse serum to stimulate migration or invasion respectively for 24 and 48 h. The cells at the bottom of the membranes were fixed with cold methanol, stained with Eosin (cytoplasm) and Thiazine (nucleus), and mounted onto glass chamber slides to count all cells. Assays were done in triplicate.

### Migration (wound-healing) assays using scratch method

Cells grown to semi-confluency were serum-starved over-night. Proliferation inhibitor mitomycin C (1 ng/μl) was added 2 h before the plate was scratched with a microtip. Cells were then incubated in medium including 1% horse serum and mitomycin C for 72 h, replacing media every 24 h. Migration, un-affected by proliferation was measured as the distance covered per unit time.

### Zymography

Cell-free conditioned media were collected at 24 h and frozen at − 20 *°*C. 10% zymogram gels were made with Gelatin A to measure gelatinolytic activity. Conditioned medium mixed 1:1 with zymogram sample buffer, was loaded and run at 110 V for 90 min. The gel was incubated in zymogram renaturation buffer for 1 h, then in zymogram developing buffer for 1 h, and finally overnight at 37 *°*C in fresh zymogram developing buffer (all from BioRad, Mississauga, ON). Gels stained with Coomassie Blue were imaged using the BioRad XR+ Gel documentation system.

### Tumorsphere (spheroid) formation assay

MCF7 and MCF10A cells were grown as spheroids on ultra-low attachment plates as reported [6]. In brief, cells grown to 70–80% confluency were trypsinized and spun down. They were then suspended in basal HuMEC media (Gibco) with added B-27 supplement (Gibco), EGF (20 ng/mL, Invitrogen) and FGF (20 ng/mL, Invitrogen), taken up by a 1 mL syringe and put through a 40 μm cell strainer (Falcon) to collect a single cell suspension. The cells were then counted and seeded at a density of 10 cells/well in an ultra-low attachment 96-well plate (Thermo Fisher) to measure spheroid forming efficiency (SFE), as well as 1 × 10^3^ cells/well in an ultra-low attachment 6-well plate (Corning, NY, USA) for for RNA analysis and immunofluorescence. SFE was calculated at 4 days as the total number of spheroids (minimum 60 μm in diameter) divided by total number of cells plated.

### Polysome profiling

Polysome profiling was performed as reported [33]. WT or CBEB2KO MCF10A cells were pre-treated with cycloheximide (CHX, 100 μg/mL) for 5 min at 37 °C. Cells were washed twice with ice-cold PBS containing 100 μg/mL CHX and lysed in hypotonic buffer (100 mM KCl, 50 mM Tris–HCl pH 7.4, 1.5 mM MgCl2, 1 mM DTT, 1 mg/mL heparin, 1.5% NP40, 100 μg/mL CHX, supplemented with mini cOmplete™ Protease Inhibitor Cocktail tablet and 100 Unit RiboLock RNase inhibitor). The lysates were incubated on ice for 5 min and cleared by centrifugation at 12000 rpm for 5 min at 4 °C, prior to loading on 10–50% sucrose gradients to isolate the sub-polysomal and polysomal fractions. Total RNA was isolated from each fraction using Trizol reagent and converted to cDNA using SuperScript III First-Strand Synthesis System (Life Technologies, Burlington, ON). RT-PCR was performed for each gene using a PCR thermocycler (Bio-Rad) and visualized by agarose gel electrophoresis.

### Cell proliferation assays

WT or CPEB2KO MCF10A cells were incubated for 24 h in complete media with or without EDU (negative control for autofluorescence) using Click-iT EdU Alexa Fluor 488 Imaging kit (Invitrogen). EdU incorporation was measured in an analytical flow cytometer. BrdU incorporation was measured in WT, MOCK and CPEB2KD MCF7 cells using the Cell Proliferation ELISA, BrdU (colorimetric) Kit (Roche, Sigma) and an Epoch Microplate Spectrophotometer at a wavelength of 370 nm with a reference wavelength of 492 nm.

### Animal experiments for tumorigenicity assays

NOD/SCID/IL2R**γ**-null (NSG) mice (deficient in T, B and NK cells) were bred locally by Dr. David Hess (co-author) and maintained according to the Canadian Council of Animal Care guidelines with food and water ad libitum at the Robarts Research Institute mouse barrier. The protocol was approved by the Animal Care and Veterinary Services (ACVS) committee on animal use. Six-week-old females were used as hosts for tumorigenicity assays by intravenous or subcutaneous routes using 5 × 10^5^ CPEB2KO and WT MCF10A cells per mouse. They were euthanized with CO_2_ at the appropriated time. Tail vein injected mice (*n* = 6) were sacrificed at 8 weeks to isolate lungs, spleen and liver to assess metastases. Cells mixed 1:1 with Matrigel were injected S.C. into both right and left inguinal mammary regions of 5 mice each for CPEB2KO or parental cells (total = 10 sites each). After 12 weeks at sacrifice, one mammary fat pad was stored in O.C.T. for frozen sectioning and the other in Bouin’s solution for paraffin embedding. Lungs, liver and spleen were also harvested to assess spontaneous metastasis from mammary sites. All lungs were inflated with PBS prior to isolation. All mice were weighed once per week.

### Tissue processing from mice

Lungs, liver and spleen were stored either in O.C.T for cryo-sectioning or Bouin’s solution/Neutral-Buffered Formalin for fixation and paraffin embedding. Paraffin blocks were sectioned at 5 μm and stained with H&E. Frozen organs were sectioned at 8 μm for lungs, liver and spleen, while mammary fat pads were sectioned at 10 μm. Sections were then fixed in 10% formalin, permeablized in 0.1% Triton-X-100, and blocked with M.O.M (mouse-on-mouse Ig, Vector labs, Burlington, ON) prior to immuno-staining. Mouse anti-HLA antibody (1:100, BD, Mississauga, ON), followed by horse anti-mouse FITC (1:200, Vector Labs) was applied to detect human cells. Sections were mounted with Vectashield (Vector Labs), stained with DAPI and viewed under a fluorescent microscope. Micrometastases were arbitrarily scored as single cells, clusters (2–8 cells) and colonies (> 8 cells) as reported earlier [6] and averaged within 3 sections, 5 images of 1600μm^2^ each per section.

### Measurements of CPEB2 expression in human breast cancer cell lines, breast cancer and non-tumor breast tissues

Using the blast search, we found that primers used by Johnson et al. [29] for CPEB2A covered all 6 isoforms, whereas primers used for CPEB2B covered both isoforms B and D. In our study we used probes with increased isoform specificity: a Taqman probe for isoform A/E and another probe for isoform B/D (Applied Biosystems, ThermoFisher) to conduct qPCR in a panel of human breast cancer tissues (*n* = 105) and non-tumor breast tissues (*n* = 20) obtained from the Ontario Tumor bank (OTB) maintained by the Ontario Institute of Cancer Research (OICR) following ethics approval by the OICR committee. This bank receives tumor tissues from donors in Ontario hospitals following donor consent. Taqman probes (Applied Biosystems) for GAPDH and β-Actin were used as the internal loading control. Delta Ct values were calculated by subtracting the average Ct values (triplicate) from the control and analyzed as previously described [6].

The probe for isoform A/E was also utilized to compare *CPEB2A* expression in a panel of *COX-2* disparate human breast cancer cell lines MCF7, MCF7-COX2, MDA-MB-231 and SKBR3. To conduct qPCR for *CPEB2* in MCF7 cells we used a Taqman probe from Life Sciences, which is not isoform-specific. To conduct RT-PCR for *CPEB2* in MCF10A cells, we designed oligos to cover all isoforms: Forward: ACACTCTTACCCTTACAGGTG; Reverse: CGCCCATAACTCCTTGCATT.

### Statistical analysis

Statistical analyses were performed using Graphpad Prism Software 5.0 (Graphpad Software Inc. 2007). All parametric data were analyzed with one-way ANOVA followed by Tukey-Kramer or Dunnett’s post hoc comparisons. Spheroid and lung colony numbers were analyzed both by parametric and nonparametric (Mann-Whitney test) methods, giving similar results. Student’s t test was used to compare two datasets. Statistically relevant differences between means were accepted at *p* < 0.05.

## Results

### CPEB2 is the single COX-2 down-regulated gene targeted by miR-526b and miR-655

We found that miR-526b and miR-655 collectively targeted a total of 13 *COX-2* -down-regulated genes, 12 of which are tumor-suppressor-like. The remaining single gene targeted by both microRNAs was identified as *CPEB2* (Additional file 4: Table S1).

### CPEB2 expression levels in multiple breast cancer cell lines

**Fig. 1:**
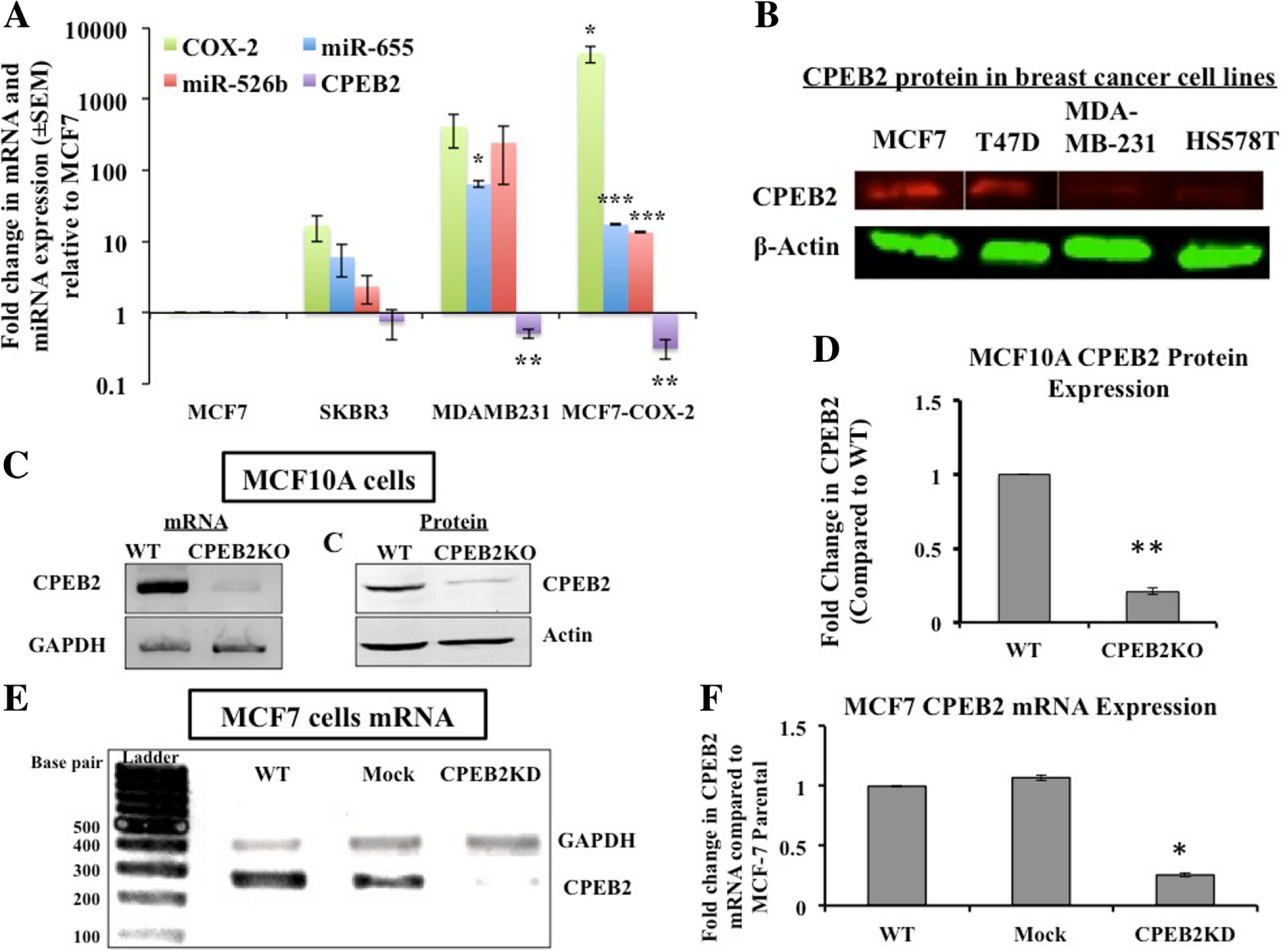
CPEB2A expression in various un-manipulated and manipulated cell lines. **a** *COX-2*, miR-655, miR-526b and *CPEB2A* were quantified in different (COX-2 disparate) breast cancer cell lines, relative to MCF7 cells. Cell lines (MDA-MB-231 and MCF7-COX-2) with high *COX-2* and miRNAs expression showed low expression of *CPEB2A* (measured with an A/E probe). **b** Western blots for CPEB2 protein (about 60 kDa, presumably CPEB2 A, identified with an isoform nonselective antibody) revealed a similar trend, high expression of CPEB2 in poorly malignant, low COX-2 expressing MCF7 and T47D cells, and low expression in high COX-2 expressing and metastatic MDA-MB-231 and Hs578T cells. **c**, **d** CPEB2 knockout (KO) through a double nickase CRISPR plasmid in MCF10A cells resulted in downregulation of CPEB2 mRNA (shown with RT-PCR) and protein (shown with Western blot). CPEB2 protein expression was knocked out with 80% efficiency. **e**, **f**. SiRNA-mediated knockdown of *CPEB2* mRNA using a pool of siRNAs in MCF7 cells shows 75% downregulation. Data presented as mean of 3 replicates ± SEM. (*) indicates *p* < 0.05, (**) *p* < 0.001, (***) *p* < 0.0002

*CPEB2* mRNA expression (of the A/E isoform) compared in multiple *COX-2* divergent cell lines (Fig. 1a) shows an approximately inverse relationship with *COX-2* and the miR-526b and miR-655 expression levels. Similarly CPEB2 protein levels (measured in western blots with an isoform-non-selective antibody, that produced a single band of putative isoform A (approx. 60 kDa) were higher in *COX-2-*low (MCF7, T47D) than in *COX-2-* high (MDA-MB-231, HS578T) cells (Fig. 1b).

### Validation of CPEB2 down-regulation in CPEB2KO MCF10A cells and CPEB2KD MCF7 cells

CPEBE2 mRNA and protein levels were very high in MCF10A cells, in which we knocked-out *CPEB2* (inclusive of all isoforms) using a double nickase CRISPR plasmid to ensure high specificity. A comparison of mRNA and protein levels in WT and KO cells (Fig. 1c) demonstrated approximately 80% knock-out efficiency (Fig. 1d). PCR data revealed a single band corresponding to isoform A, both in WT and CPEB2KO MCF10A cells confirming that MCF10A cells lack in the B isoform [29]. We also knocked down *CPEB2* in the poorly malignant, CPEB2A dominant breast cancer cell line MCF7, using a pool of siRNAs. Scrambled siRNAs served as controls (Mock cells). An efficient knock-down (approximately 75% relative to WT or Mock cells) was noted at the mRNA level (agarose gel picture in Fig. 1e; quantification of Fig. 1e presented in Fig. 1f).

### CPEB2 downregulation in breast epithelial cells induces EMT and promotes migration and invasion

**Fig. 2:**
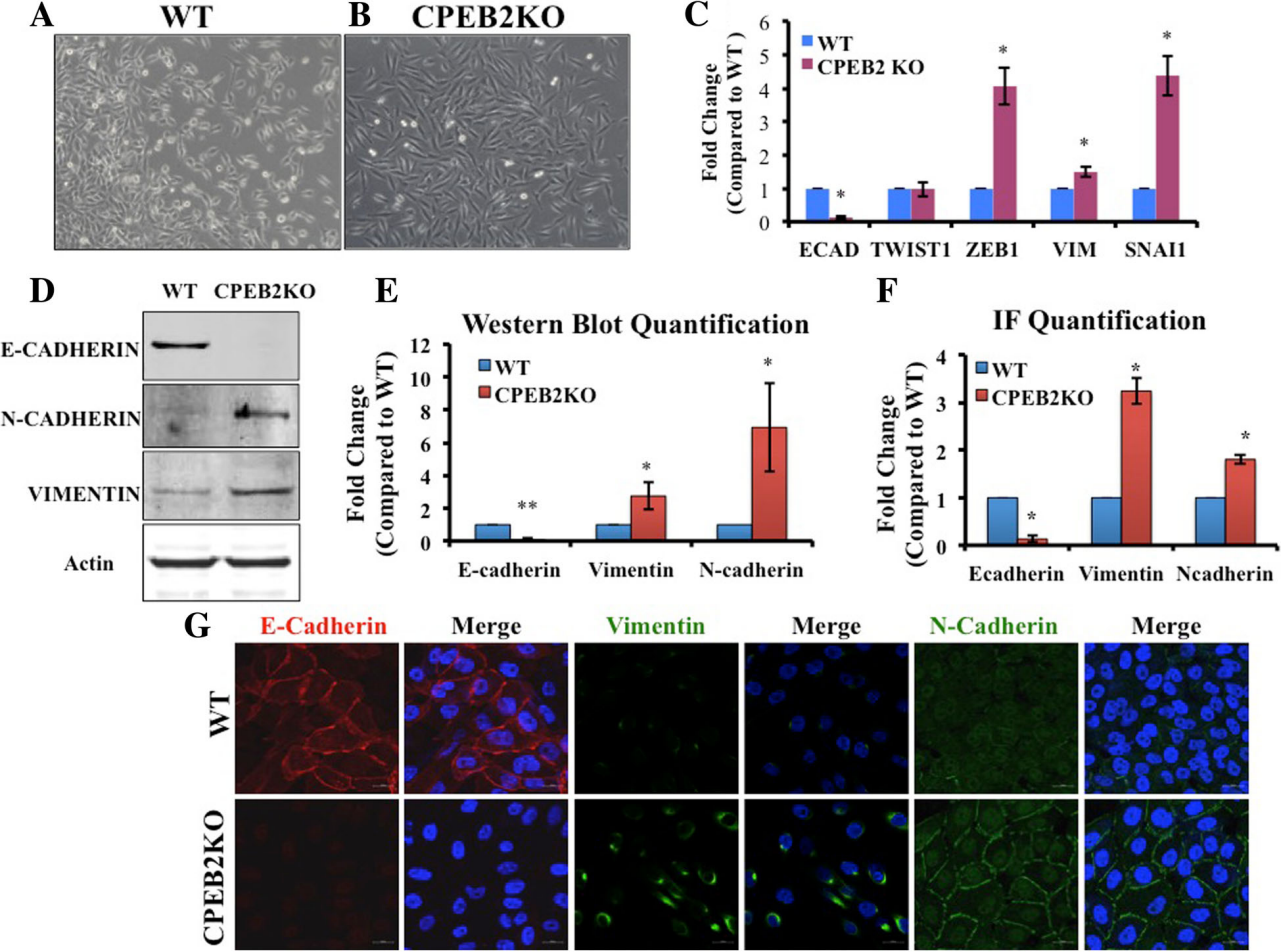
Epithelial to mesenchymal transition in CPEB2KO cells. **a** Wildtype (WT) MCF10A cells exhibiting epithelial cell morphology. **b** CPEB2KO cells exhibiting elongated, mesenchymal (Fibroblast-like) morphology (Magnification viewed at10x objective). **c** Quantitative RT-PCR for EMT marker mRNAs in WT and CPEB2KO MCF10A cells. Epithelial marker E-Cadherin was significantly decreased (to 0.116 fold), with significant increases in mesenchymal markers ZEB1 (4.06 fold), Vimentin (1.49 fold), and SNAI1 (4.38 fold) in CPEB2KO cells. **d**, **e** Induction of EMT in CPEB2KO cells shown at the protein level. Representative Western blots (**d**) and quantification of Western Blots (**e**) for E-Cadherin, Vimentin and N-Cadherin in wildtype and CPEB2KO cell lines showing significantly decreased E-Cadherin protein (to 0.078 fold), significantly increased Vimentin (to 2.75 fold) and N-Cadherin (to 6.93 fold) in CPEB2KO cells. **f**, **g** EMT visualized through immunofluorescence of markers. **g**. E-Cadherin (red), Vimentin (green), and N-Cadherin (green); Nuclei (blue). Magnification Scale = 20 μm. **f**. Integrated fluorescence density quantified with ImageJ Software and normalized to cell number, showing that E-Cadherin protein expression was significantly decreased (to 0.20 fold), with a significant increase in Vimentin (to 3.50 fold) and N-Cadherin (to 1.80 fold) in CPEB2KO cells compared to WT cells. Data presented as mean of 3 replicates ± SEM. (*) indicates *p* < 0.05, (**) *p* < 0.001

Upon *CPEB2* knock-out, the epithelial-like (polygonal shaped) wild-type MCF10A cells (Fig. 2a) assumed a mesenchymal-like (spindle-shaped) morphology (Fig. 2b). To test an association of this morphological change with EMT, we compared EMT markers in WT and CPEB2KO cells using qRT-PCR and Western blots. mRNA expression for the epithelial marker *CDH1* (E-Cadherin, a transmembrane protein) was suppressed, with a concomitant increase in mesenchymal markers *VIM* (Vimentin, an intermediate filament) and two transcription factors *SNAI1* and *ZEB1* (Fig. 2c). Western blots revealed a near-complete depletion of E-Cadherin and increases in mesenchymal markers N-Cadherin and Vimentin proteins (Fig. 2d and e). Cellular immunofluorescence for the respective proteins corroborated this phenotype of reduced E-Cadherin, increased N-Cadherin and Vimentin (Figs. 2g showing morphology, and 2F quantitation). Similarly, CPEB2KD MCF7 cells also displayed EMT phenotype, as illustrated by a significant down-regulation of E-Cadherin and upregulation of Vimentin and TWIST proteins relative to WT or Mock-transfected MCF7 cells, identified with immunofluorescence and Western blots (Additional file 1: Figure S1).

**Fig. 3:**
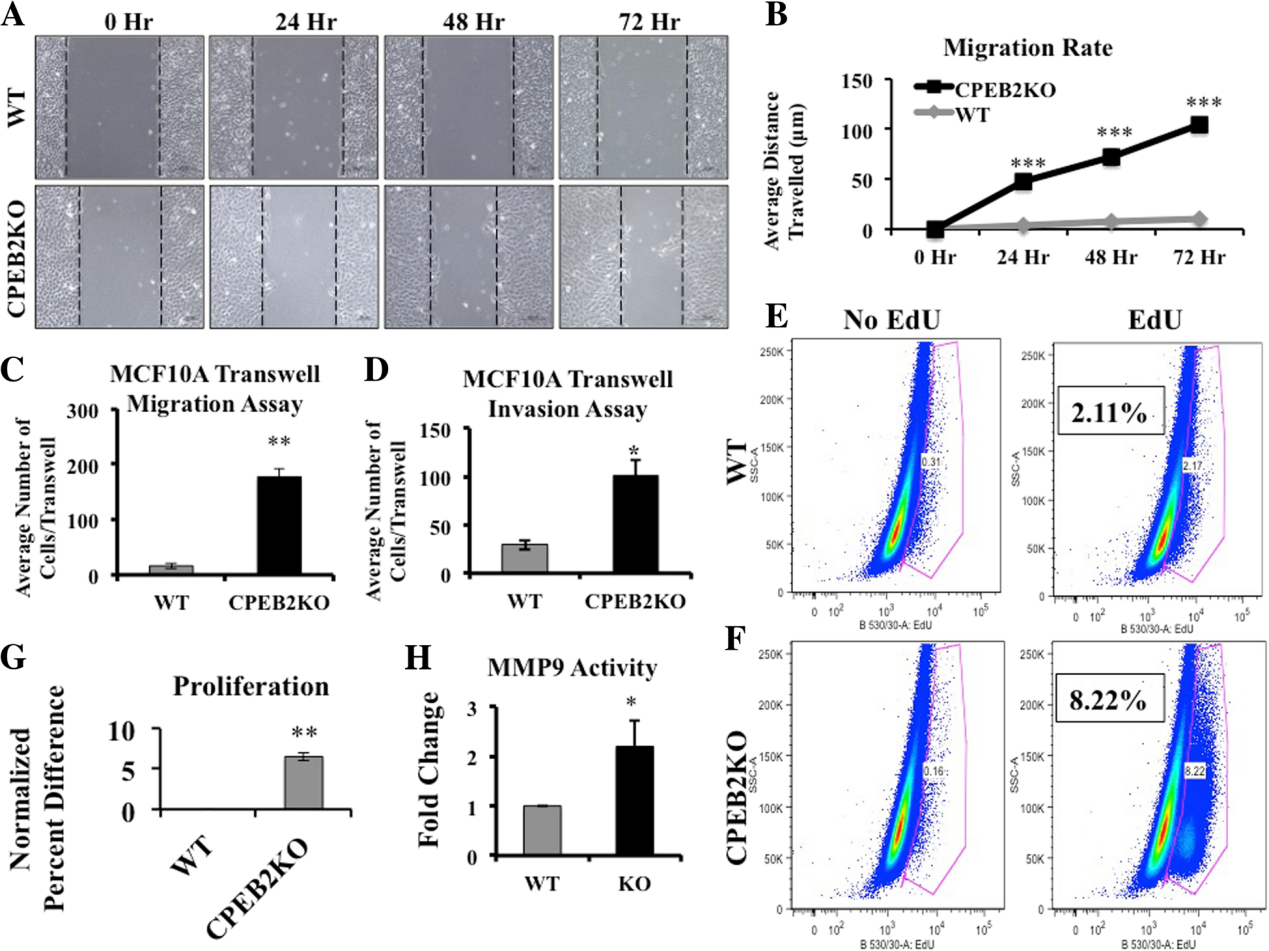
Comparison of migration, invasion and proliferation in WT and MCF10A CPEB2KO cells. **a** Representative images of WT and CPEB2KO MCF10A cells that were scratched and allowed to migrate in 1% Horse Serum, in the presence of mitomycin C to block proliferation. **b** Cell migration rate was measured as (distance at 0 h – distance at 24 h or 48 h or 72 h). WT cells migrated in a rate of 3.57 μm/day, whereas CPEB2KO cells migrated 35.82 μm/day. Scale = 100 μm. **c** Migration and (**d**) invasion of WT and CPEB2KO MCF10A cells in transwell chambers. Serum starved cells were allowed to migrate/invade in the presence of 5% Horse Serum in the bottom chamber. Cells were treated with Mitomycin C to block proliferation. The migration rate was 10.89 fold and invasion 3.43 fold in CPEB2KO cells relative to WT cells. **e**, **f** Proliferation (24 h EdU uptake measured with flow cytometry for immune-fluorescence) showing about 4 fold increase in CPEB2KO cells relative to WT cells in a representative assay. **g** Data presented as mean normalised % difference in proliferation between KO and WT cells (KO-WT)/WT. **h** Gelatinase A (MMP9) activity in WT and CPEB2KO MCF10A cells measured with densitometry of zymograms, WT cells normalised to 1. Quantitative data presented as mean of 3 ± SEM. (*) indicates p < 0.05, (**) p < 0.001, (***) *p* < 0.0005

EMT is a functional associate of cellular migratory and invasive abilities due to changes in molecular expression profiles. For example, loss of E-Cadherin emancipates cells from contact inhibition [34, 35], whereas an increase in Vimentin mediates cytoskeletal reorganization needed for motility [36]. To assess changes in migration alone independent of proliferation, cells were serum-starved overnight and incubated with mitomycin C prior to scratching (or wound-healing) assay. CPEB2KO cells migrated at a significantly faster rate than wild-type cells during 72 h (Fig. 3a and b). By 24 h, the migration was 10-fold faster.

Migration was also measured in transwells (chemokinesis assay), in which serum-starved cells were allowed to migrate through microporous membranes for 24 h into a medium containing 5% Horse Serum. CPEB2KO cells migrated faster than WT cells (Fig. 3c). Similarly, CPEB2KD MCF7 cells also migrated faster than the Mock (scrambled siRNA-transfected) or WT MCF7 cells (Additional file 2: Figure S2A).

Ability to invade basement membrane components is an important prerequisite for metastasis. Invasion was measured as above for 48 h, in which the microporous membranes were coated with a basement membrane analog Matrigel. CPEB2KO MCF10A cells exhibited a higher invasive ability than WT cells (Fig. 3d). CPEB2KD MCF7 cells also showed significantly higher invasiveness than Mock or WT MCF7 cells (Additional file 2: Figure S2B). Increased matrix-degrading ability of CPEB2KO MCF10A cells was corroborated by gelatin zymography, showing a 2.9 fold increase in gelatinase A (MMP-9) activity (Fig. 3h).

### CPEB2KO promotes proliferation

Sustained proliferative ability is a hallmark of cancer cells [37]. Flow cytometry for 5′- ethynyl-2′-deoxyuridine (EdU) incorporation (measuring DNA synthesis) for 24 h revealed a four-fold increase in CPEB2KO MCF10A cells compared to WT cells (Fig. 3e and f) in 3 replicate preparations. CPEB2KD MCF7cells, however, exhibited only a minor increase (*p* = 0.06) in proliferative ability compared to mock cells (Additional file 2: Figure S2C).

### P53 translation is repressed in CPEB2-KO cells

**Fig. 4:**
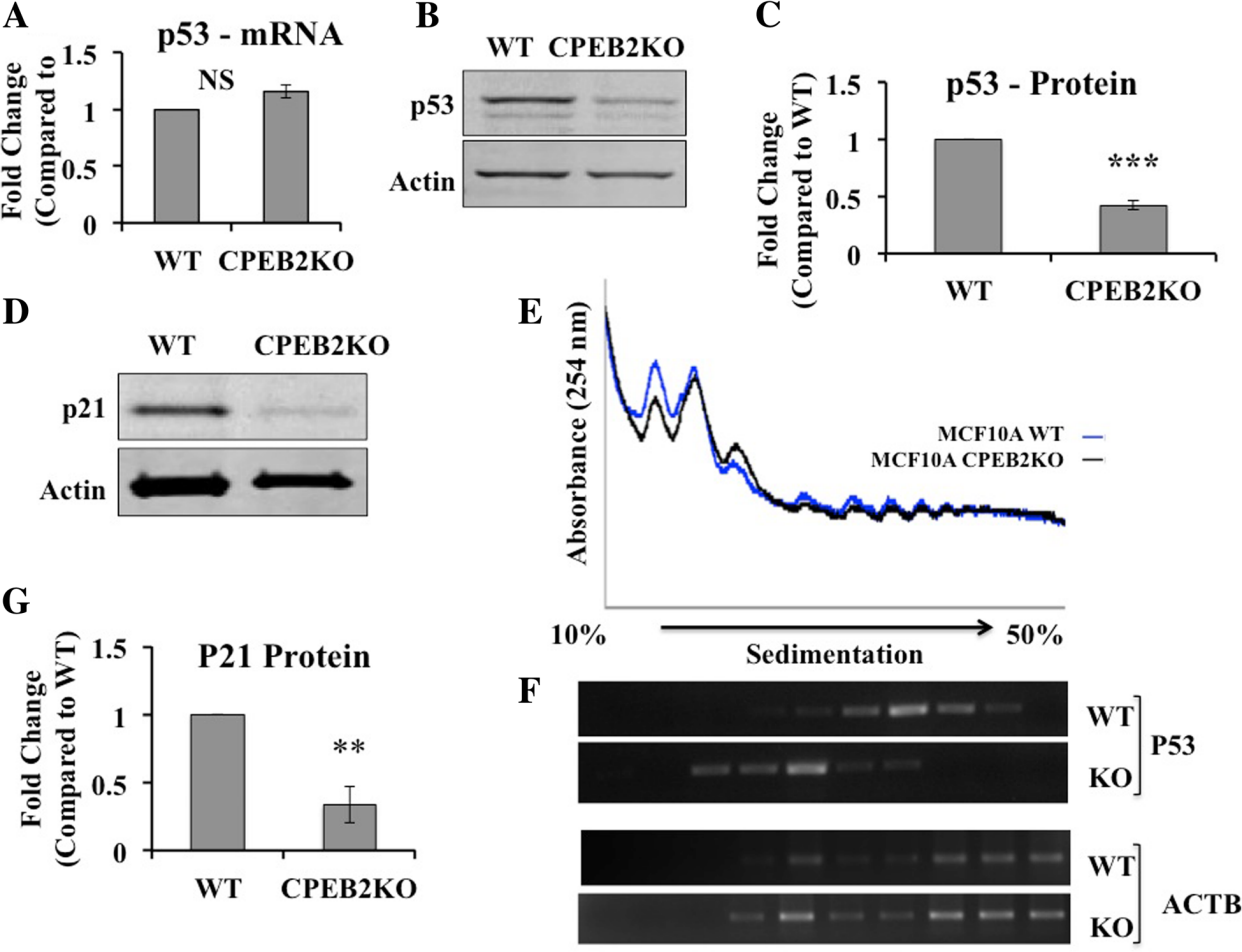
Translational regulation of p53 in CPEB2KO cells. **a)**qRT-PCR for p53 mRNA expression showing no difference between WT and CPEB2KO cells. **b** Western Blots for p53 and (**c**) quantification of western blots showing p53 protein expression was significantly reduced (to 0.41) in CPEB2KO cells. **d**, **g** p21 (downstream effector of p53) protein expression was significantly reduced (to 0.34) in CPEB2KO compared to WT cells. Western blot presented in (**d**) and quantitation in (**g**). Data represent mean of 3 replicates ± SEM. (**) indicates p < 0.001, (***) *p* < 0.0001. **e** Polysomal profiles of global protein translation in WT and CPEB2KO MCF10A cells showing no difference. **f** RT-PCR was performed to evaluate the distribution across a sucrose density gradient of ACTB and p53 mRNAs, showing that p53 mRNA was significantly shifted toward light polysomes in CPEB2KO cells

CPEB1 and CPEB2 were reported to co-regulate two transcription factors HIF1α [24] and TWIST1 [27]. CPEB1 was also shown to be a translational regulator of p53, a powerful tumor- suppressor. *P53* mRNA contains 2 CPE domains in its 3′ UTR, which promote polyadenylation. In CPEB1 knock-down cells, *p53* mRNA had an abnormally short poly (A) tail reducing translational efficiency and a marked decrease in p53 protein [38]. Hence using CPEB2KO MCF10A cells we examined whether p53 is a candidate translational target of CPEB2. qRT-PCR revealed no significant difference in *p53* mRNA expression between CPEB2KO and WT cells (Fig. 4a). However, p53 protein levels measured with western blot, revealed ∼60% reduction (Fig. 4b and c), suggesting that p53 is differentially regulated at the translational level. We also examined expression of p21 protein, a downstream effector of p53, using western blot and found a 66% reduction in the CPEB2KO cells (Fig. 4d and g), indicating that the p53 pathway is negatively affected.

To interrogate whether p53 protein was translationally regulated by CPEB2, we compared polysome profiling between WT and CPEB2KO MCF10A cells. The profile appeared almost identical indicating no significant difference in global translation between the two cell types (Fig. 4e). RT-PCR was performed to evaluate the distribution of *β-actin* and *p53* mRNAs across a sucrose density gradient. While *β-actin* distributions were similar in both cells, *p53* mRNA was significantly shifted toward light polysomes in CPEB2KO cells (Fig. 4f). These results, combined with the findings of the absence of any change in *p53* mRNA in CPEB2KO cells, clearly reveal that *CPEB2* knock-out decreased the translation of *p53* by shortening the Poly A tail leading to decreased p53 protein.

### CPEB2KO/KD stimulates SLC phenotype

**Fig. 5:**
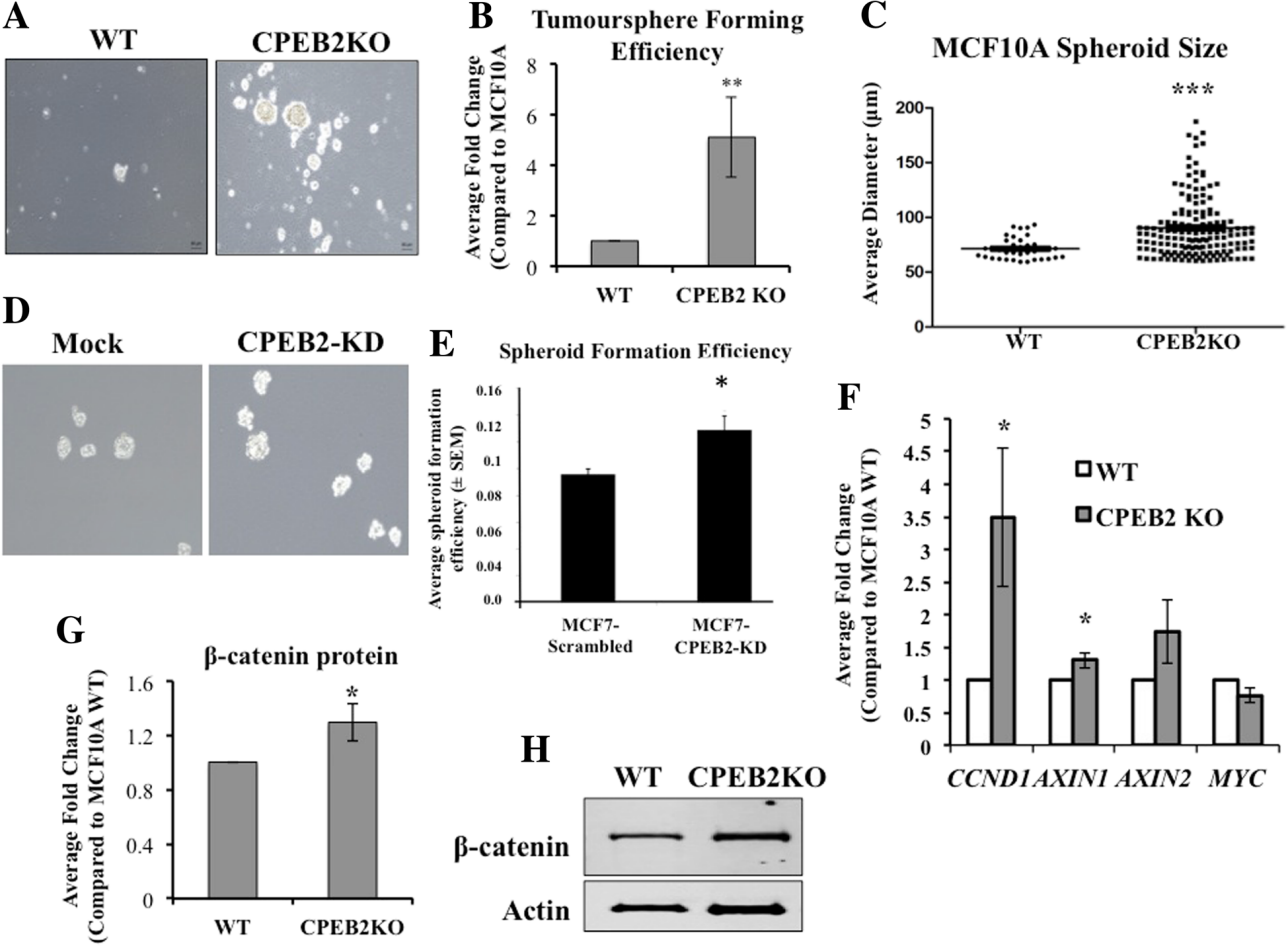
Increased spheroid formation by CPEB2-KO and CPEB2-KD cells. **a** Representative images of spheroids (Scale = 60 μm) and (**b**) spheroid forming efficiency (SFE) of WT and CPEB2KO MCF10A cells grown on ultra-low attachment plates for 4 days. SFE is computed as the number of spheroids (> 60 μm) divided by total number of cells plated. CPEB2KO cells showed 5.12-fold increase in SFE (*p* < 0.001). **c** Dot plot of spheroid size (Mann-Whitney Test for statistical significance) showing increased average diameter (WT = 70.92 μm, CPEB2KO = 91.42 μm; p < 0.0005), indicating enhanced spheroid growth rate. **d** Images of spheroids and (**e**) SFE in Mock and CBEB2-KD MCF7 cells, showing an increase in CPEB2-KD cells. **h** Representative Western blot and (**g**) quantification (Mean of 3 ± SEM) of β- Catenin protein expression. **f** qRT-PCR (Mean of 3 ± SEM) for downstream genes of β-Catenin pathway. β-Catenin was increased 1.29 fold (*p* = 0.048) in CPEB2KO cells, with increases in downstream target genes CCND1 (3.49 fold, *p* = 0.039), and AXIN1 (1.298 fold, *p* = 0.034). No significant change was observed in AXIN2 (1.73 fold change, *p* = 0.10) or Myc (0.76 fold change, *p* = 0.051)

CPEB2 being a common target of both SLC-promoting miRNAs, we examined whether *CPEB2* downregulation in MCF10A and MCF7 cells stimulated SLC properties using the spheroid (tumorsphere) formation assay which measures the ability of single cells to self-renew in an anchorage-independent manner when grown on ultra-low attachment plates [6]. CPEB2KO MCF10A cells displayed significantly increased spheroid formation (morphology shown in Fig. 5a) and spheroid forming efficiency (SFE, shown in Fig. 5b) as well as growth rate of spheroids (indicated by the spheroid size) on day 4, compared to WT cells (Fig. 5 C). CPEB2KD MCF7 cells also displayed significant increase in spheroid formation (morphology shown in Fig. 5d and SFE in Fig. 5e).

**Fig. 6:**
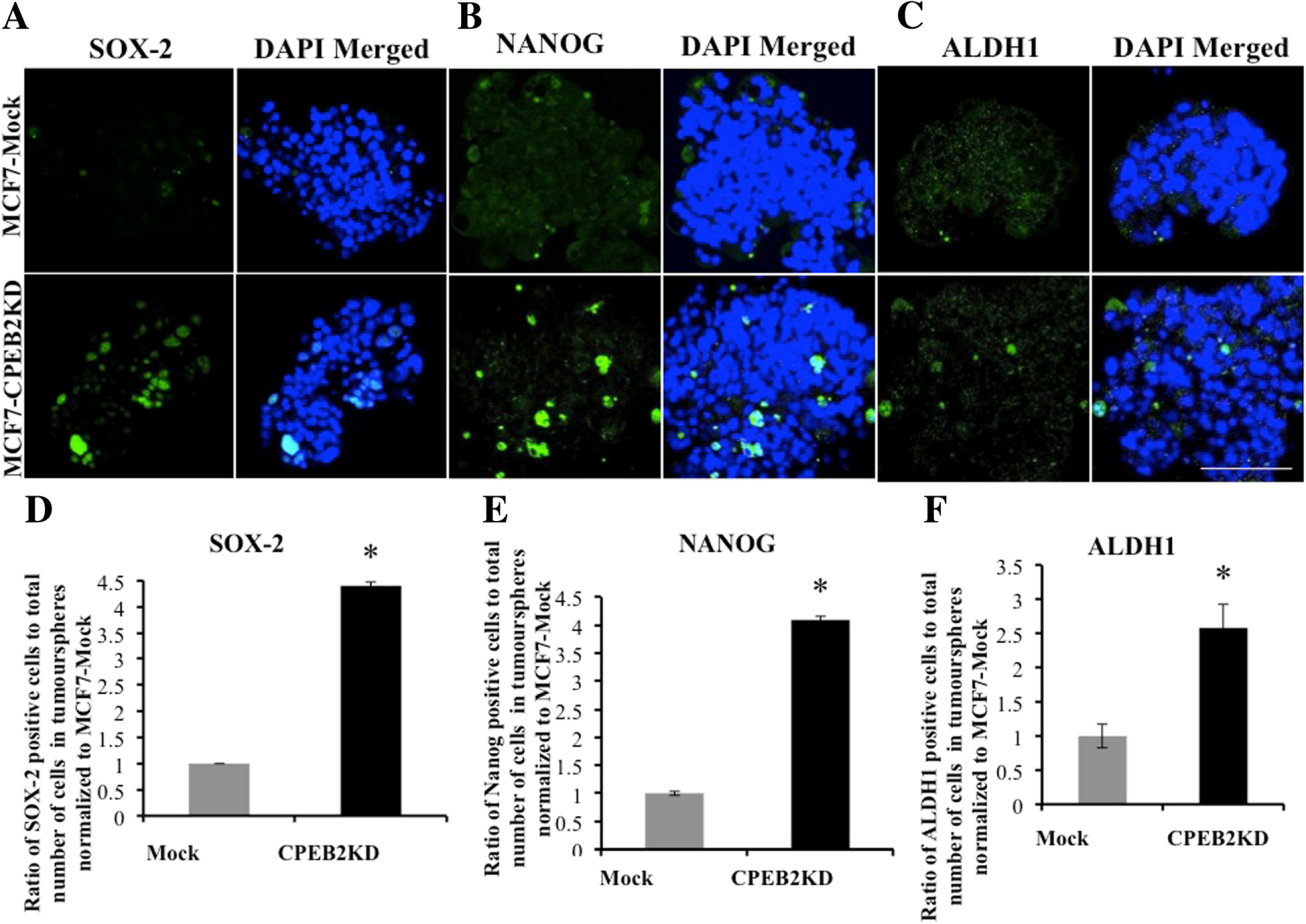
SLC markers in Mock and CPEB2KD MCF7 spheroids. Immunofluorescence images for SLC markers (**a**) SOX2, (**b**) NANOG and (**c**) ALDH1 in green, nuclei stained blue with DAPI in spheroids, (scale = 50 μm). Quantitative data for SLC markers, (**d**) for SOX2, (**e**) for NANOG and (**f**) ALDH1 showing an increase in markers bearing cells in CPEB2-KD spheroids compared to MCF7-Scramble spheroids. Data presented as mean of 3 replicates ± SEM (*) indicates *p* < 0.05

Wnt/β-Catenin signaling is a well-known pathway used by cancer cells for multiple malignancy-associated functions including SLC properties [39], as demonstrated in *COX-2* over-expressing breast cancer cells [6]. CPEB2 was reported to bind *β*-Catenin mRNA to repress translation in mouse neuronal cells [40]. Therefore we compared the levels of β-Catenin protein in WT and CPEB2KO MCF10A cells, as well as changes in the expression of genes (*AXIN2, AXIN1, CCND1, cMyc*) downstream in the canonical Wnt/β-Catenin pathway. Western blot for β-Catenin protein revealed an increase in CPEB2 KO compared to WT cells (Fig. 5g and h). This was associated with significant increases in *CCND1* and *AXIN1* mRNAs (Fig. 5f), suggesting that Wnt/β-Catenin pathway may be involved in SLC stimulation noted earlier. We also immuno-stained spheroids for certain SLC-associated markers, as reported earlier [6]. CPEB2KD MCF7 cells showed significantly increased incidence of cells expressing SOX2, NANOG and ALDH1, compared to control mock-transfected MCF7 cells (Fig. 6).

### CPEB2KO MCF10A cells reveal an upregulation of VEGF-D, COX-2 and EP4

We reported that ectopic *COX-2* over-expressing MCF7 cells displayed an upregulation of VEGF-A, VEGF-D and EP4 receptor [6]. Furthermore two COX-2-upregulated miRNAs miR-526b and miR-655, which target *CPEB2*, when ectopically over-expressed in miRNA-low MCF7 cells led to an upregulation of EP4 and COX-2, indicating a positive feed-back loop for pathways in SLC sustenance [11, 12]. These findings prompted us measure *VEGF, EP4* and *COX-2* mRNAs in CPEB2KO MCF10A cells. We found a significant upregulation of *VEGF-D, COX-2* and *EP4* mRNAs (Additional file 3: Figure S3). Conversely, inhibition of COX-2 or EP4 activity in MCF7-COX2 cells which was found to suppress SLC activity [6] also upregulated *CPEB2* (data not presented).

### CPEB2KO MCF10A cells form tumors in immune-compromised mice

**Fig. 7:**
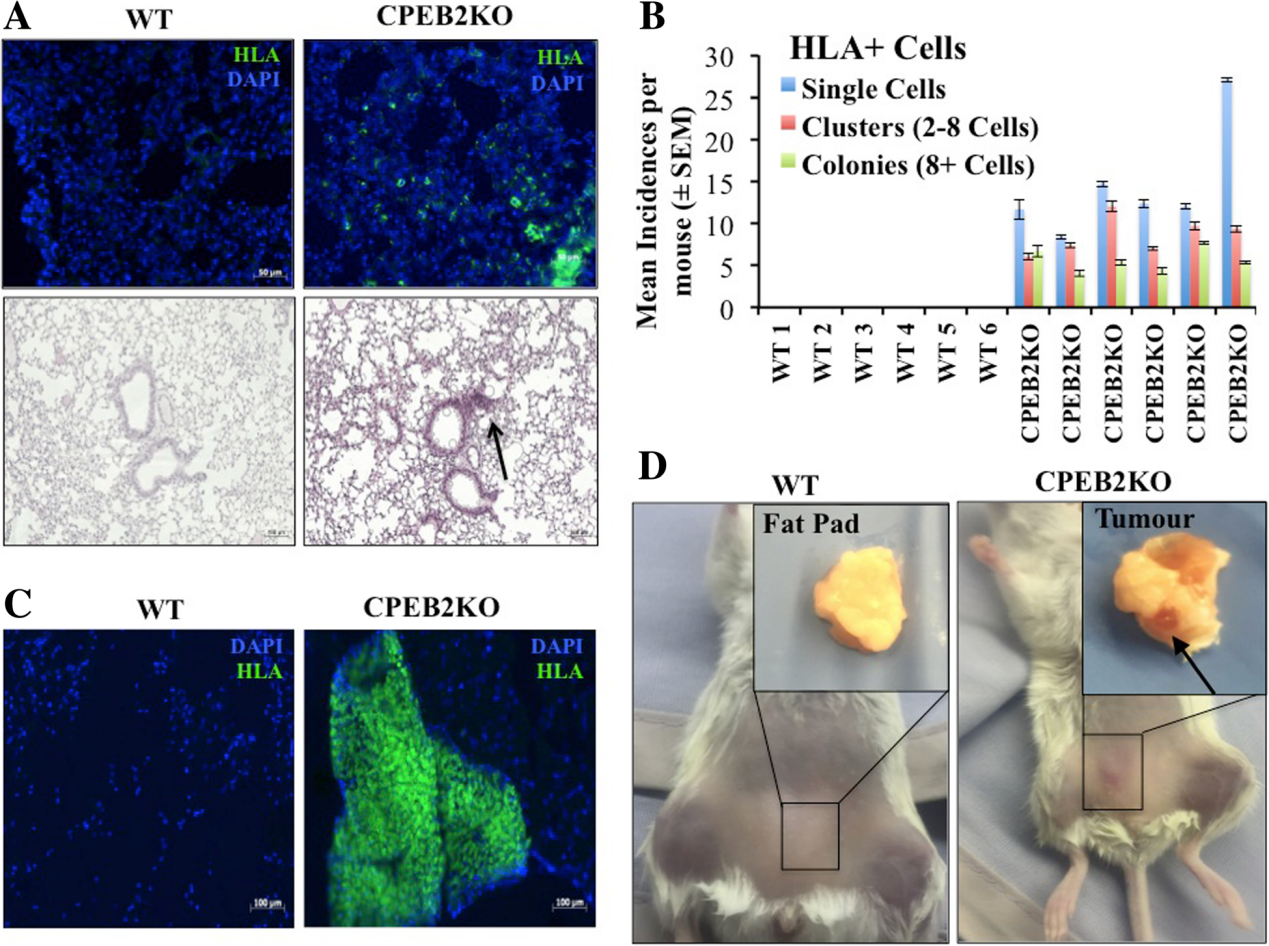
Tumorigenicity of WT and CBEB2KO MCF10A cells in NOD/SCID/IL2Rγ null mice. **a**, **b** Intravenous injection of cells (5 × 10^5^ cells per mouse, *n* = 6 mice per cell line) resulted in lung metastasis of CPEB2KO but not WT cells. **a** Top panel: HLA stained (green) tumor cells (single cells, clusters and colonies) noted in the lungs with CPEB2KO inocula. Nuclei stained blue with DAPI. Bottom panel: H&E stained lung showing a small tumor-like lesion (pointed with arrow) with CPEB2KO inoculam. Scale = 50 μm (IF images) and 100 μm (H&E images). **b** Incidence of single tumor cells, clusters (2–8 cells) and colonies (more than 8 cells) within lung sections immunostained with HLA antibody. Tumor cells were identified in the lungs of all mice inoculated with CPEB2KO cells but none of the lungs in mice inoculated with WT cells. Data presented as mean of 5 images per section, 3 non-serial sections per mouse ± SEM. **c**, **d** Subcutaneous inocula (5 × 10^5^ cells per site, 2 inguinal mammary sites per mouse, mixed with Matrigel, *n* = 5 mice in each group) of WT and CPEB2KO cells at the mammary sites of NOD/SCID/IL2Rγ-null mice. CPEB2KO cells formed local tumours in all mice, some of which (2 out 5, 40%) spontaneously metastasized to the lung. No tumor resulted in any mouse from WT cells. Tumour-forming efficiency was 90%, as calculated by number of sites showing local tumours (nine) divided by total number of injection sites (ten). Each mouse showed one or two tumors: 4 with double and 1 with single tumor. **d** Representative images of mice and mammary fat pads at 12 weeks. Arrow pointing to a tumor. **c** Representative images of tissues from sites inoculated with WT or CPEB2KO MCF10A cells, immunostained with HLA antibody. Tumor cells (identified only with CPEB2KO cells) stained green, and nuclei stained blue with DAPI

Using NOD/SCID/IL2Rϒ-null mice, we confirmed the findings that wildtype MCF10A cells, although immortalized, are epithelial in nature and non-tumorigenic [31, 41]*.* Intravenous inoculation of WT cells resulted in no identifiable lung colonization by gross or histological examination (H &E staining) or staining for HLA at 8 weeks. However, CPEB2KO cells formed micro-metastasis-like lesions in the lungs identified with H&E staining and staining for HLA (Fig. 7a), which could identify single cells, clusters and colonies. Quantitative data at 8 weeks are provided in Fig. 7b. Furthermore, macroscopic tumours at the mammary sites (illustrated in Fig. 7d) were noted after subcutaneous injection of the CPEB2KO cells in all mice at 9 out of 10 injection sites, but none with wild-type cells, validated with immunostaining for HLA at 12 weeks (illustrated in Fig. 7c). At 12 weeks, some of the former mice (2 out of 5) displayed spontaneous metastases to the lungs, identified with the HLA marker. Average incidence per lung sections was (400 μm)^2^ was: CPEB2KO-1: 15 +/− 2 single cells, 10+/− 2 Clusters, and 4+/− 1 colonies; CPEBE2 KO-2: 12+/− 2 single cells, 8+/− 2 clusters and 3+/− 1 colonies. None of the mice injected with WT cells displayed any lung metastasis.

### CPEB2 isoforms a and B expression in human breast cancer tissues

**Fig. 8:**
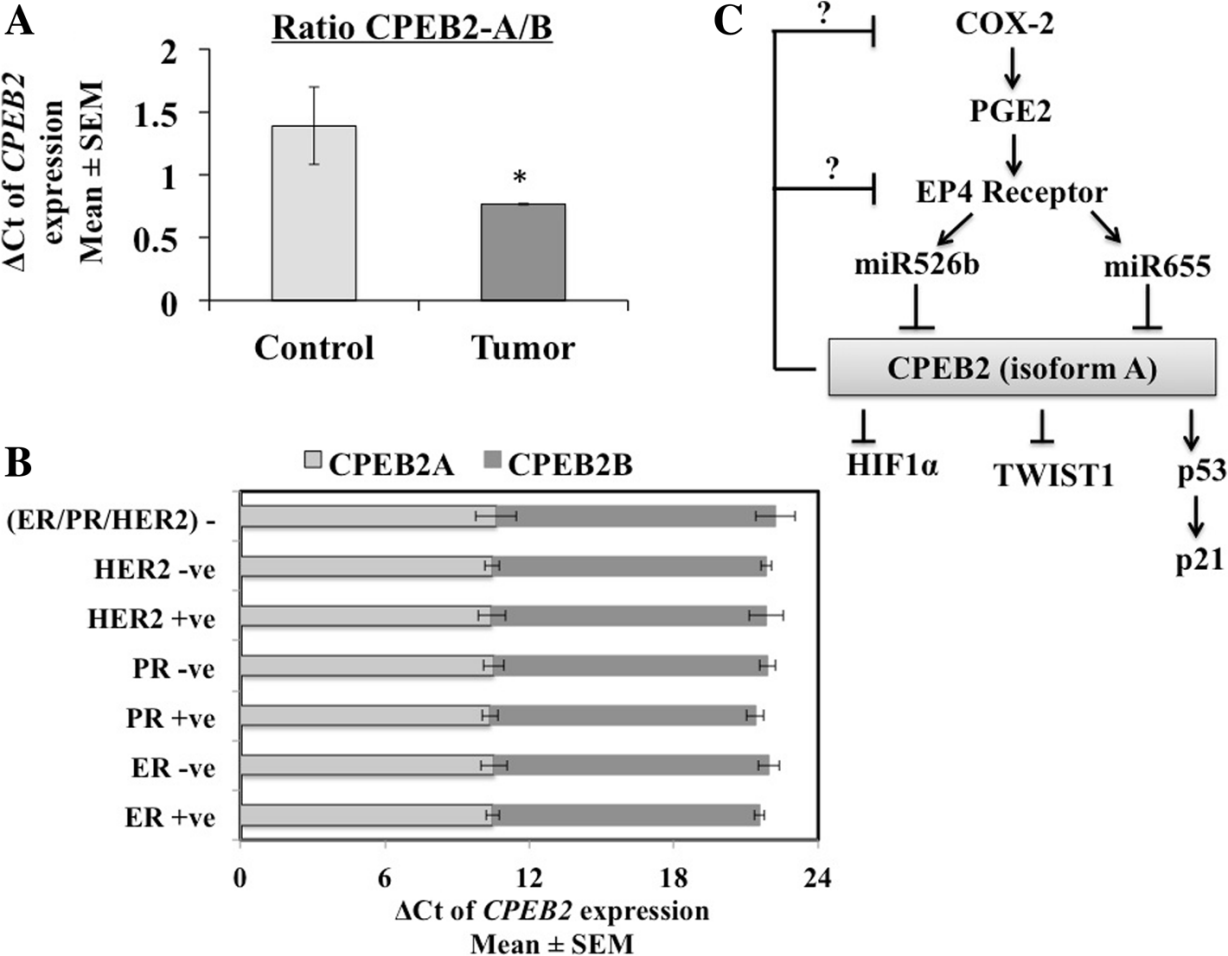
Expression of CPEB2 isoforms A and B in human breast cancer tissues and non- tumor breast tissues. Expression of CPEB2 isoforms A (measured with an A/E probe) and B (measured with a B/D probe) mRNAs were analyzed by qPCR in105 breast cancer tissues (6 samples failing to amplify expression of *GAPDH* or *CPEB2A* were removed from the study) and in 20 control (histologically tumor-free) breast tissues. **a** The control non-tumor tissues expressed relatively higher expression of isoform A, and lower expression of isoform B than tumor tissues, when normalized for *GAPDH*. They are plotted as A/B ratios of the delta Ct ± SEM. * indicates *p* < 0.05. **b** We measured *CPEB2A* (with an A/E probe) and *CPEB2B* (with a B/D probe) mRNA in various tumor subsets, ER+, ER-, PR+, PR-, HER2+, HER2-, ER/PR/HER2- normalized to GAPDH. Data presented as delta Ct ± SEM. No significant difference in the expression of A vs B was noted in any subset. **c** A schema for molecular partners in CPEB2 regulation and action in breast cancer. COX-2 via EP4 activation upregulates two oncogenic miRNAs miR-526b and miR-655, both of which target CPEB2. Tumor suppressor functions of CPEB2 (resulting from the isoform A) are mediated by multiple partners: translational regulation of p53, HIF-1α and Twist-1 mRNAs. In addition CPEB2 appears to suppress COX-2/EP4 expression by hitherto unknown mechanisms

An examination of *CPEB2* expression in TNBC was reported be isoform-selective, having increased B/A ratio [29]. We examined the expression of CPEB2 isoforms A (indistinguishable from E, by the available probe) and B (indistinguishable from D, by the available probe) in 105 breast cancer and 20 histologically confirmed non-tumour breast tissues (Fig. 8). Compared to non-tumor breast tissues, cancerous tissues exhibited a lower expression of isoform A and a higher expression of isoform B, as indicated by ΔCT, lower values indicating higher expression. The ratio of A/B isoforms (ratios of mean of ΔCT) was higher in non-tumor tissues (Fig. 8a), supporting the reports that A is the tumor-suppressor isoform and B the tumor-promoter isoform [29, 30]. The information on ER, PR and HER2 status (n value) was available in 98 out of 105 samples as follows: 19 HER2+, 64 HER2-, 75 ER+, 18 ER-, 64 PR+, 29 PR-, 11 ER/PR/HER+ and 10 ER/PR/HER2-. A comparison of CPEB2A and CPEB2B expression in different tumor subsets revealed no significant difference in any subset in our samples (Fig. 8b).

## Discussion

The roles of CPEB2 in human breast tumorigenesis have so far remained a paradox, until isoform-specific roles were identified [29, 30]. Selecting cells from TNBC cell lines for anioikosis-resistance, these authors observed that alternative splicing resulting in the loss of CPEB2A with concomitant increase in CPEB2B was responsible for their metastatic phenotype. CPEB2 was shown to suppress the translation of two oncogenic transcription factors, TWIST1 [27] - an EMT inducer, and HIF1α [24] associated with many oncogenic functions [24, 25]. Deligio et al. [30] reported that anoikosis-resistance and metastasis phenotype of TNBC cell lines resulted from CPEB2B isoform mediated translational activation of HIF1α and TWIST1. They suggested that CPEB2B plays an antagonistic role against CPEB2A by alleviating the translational inhibition of HIF1α and TWIST1 imparted by CPEB2A.

In the present study, by depleting the entire *CPEB2* gene, we demonstrated a robust role of *CPEB2* in suppressing a variety of oncogenic functions in both MCF10A and MCF7 cell lines, evidently due to the CPEB2A isoform, prevalent in both cell lines. Reportedly, no alternative splicing of CPEB2 or presence of isoform B was detectable MCF10A cell line [29]. In both cell lines, we found that *CPEB2* downregulation promoted EMT, proliferation, migratory and invasive functions, as well as SLC phenotype measured with spheroid-forming ability. The spheroids formed by CPEB2KO MCF10A cells exhibited a higher growth rate, indicating a faster self-renewal of the SLC population. Increased β-Catenin protein noted in CPEB2KO cells may be responsible for a higher self-renewal capacity of stem-like cells or increased proliferative ability of non-stem cells. For example, increased β-Catenin signaling can lead to increased expression of *CCND1*, a nuclear protein, that forms a complex with CDK4 and CDK6 leading to progression of cells from G1 into S-phase [42].

During EMT, polar epithelial cells assume an elongated nonpolar mesenchymal morphology, associated with an acquisition of mesenchymal cell markers, increased migratory ability, cell survival, and invasiveness [43]. E-Cadherin, an epithelial cell junction-associated protein mediates cell-cell adhesions, contact inhibition and control of cell proliferation [34]. It is lost during EMT [35], as shown with CPEB2KO or KD cells. However, EMT also requires “Cadherin switching” in which expression of E-Cadherin is exchanged for other cadherins, such as N-Cadherin [44, 45]. Ectopic over-expression of N-Cadherin in MCF7 cells stimulated migration and invasiveness through upregulation of MMP-9 and metastasis [46]. Here, in CPEB2KO MCF10A cells, a switch from E-Cadherin to N-Cadherin was clearly evident. Increased MMP-9 activity shown with gelatin zymography explained their increased invasive capacity. While transcriptional regulation of N-Cadherin during cadherin switching is unknown, Twist1 can modulate N-Cadherin expression by binding to the E-box on *CDH2* (N-Cadherin) [45, 47].

Numerous transcription factors may have contributed to the EMT phenotype in CPEB2KO cells. TWIST1 can mediate EMT [48], and also inhibit apoptosis through evasion of p53-induced cell death [49]. CPEB2 mediated translational repression of TWIST1, a function ascribed to the A isoform [30] could be a mechanism by which EMT is suppressed. In support, in CPEB2KO cells exhibited no change in *TWIST1* mRNA expression, suggesting post- transcriptional regulation by CPEB2. An increase in *SNAI1* and *ZEB1*, observed in CPEB2KO cells, could additionally contribute to EMT. Both molecules are known to suppress transcription of E-Cadherin and play important roles in invasion and metastasis [50, 51].

P53 is a master tumour-suppressor, responsible for suppressing EMT, migration, and invasion through transcriptional regulation of many other molecules [52]. Here, we show for the first time that CPEB2 is a novel translational regulator of *p53*, as was reported for CPEB1 [38]. Polysome profiling confirmed that a reduction in p53 protein in CBEB2KO cells is due to reduced *p53* mRNA translation resulting from shortened Poly A tail, rather than an indirect mechanism by binding to another target molecule. Additionally, another tumor suppressor protein p21, a downstream partner of p53 was also reduced in CPEB2 KO cells.

We have shown that ectopic COX-2 overexpression in MCF7 cells promoted SLC phenotype associated with increased expression of EP4 receptor, *Notch* and *Wnt* [6]. Wnt pathway protein β-Catenin and the downstream Wnt pathway genes *CCND1*, *AXIN1* and *AXIN2* were all significantly upregulated in MCF7-COX2 spheroids [6]. In the present study CPEB2KO cells exhibited an upregulation of β-Catenin protein, *AXIN1* gene, and interestingly also *COX-2* and *EP4* genes linking with SLC phenotype [6]. It is likely that an upregulation of COX-2/EP4 provided a positive feed-back loop for SLC sustenance in CPEB2KO cells, as shown for miR-526-B and miR-655 overexpressing cells [11, 12]. Conversely, treating MCF7-COX2 cells with either a COX-2 inhibitor celecoxib or with an EP4 antagonist ONO-AE3–208 significantly up-regulated *CPEB2* in these cells (data not presented), concomitant with a marked drop in their spheroid-forming capacity [6]. The molecular mechanisms underlying this CPEB2-COX2/EP4 regulatory loop remains to be investigated. One possible pathway is binding of CPEB2 to HIF-1α [24], a known upregulator of COX-2. Furthermore, increased β-Catenin protein noted in CPEB2KO cells can stabilize COX2 mRNA by interacting with AU-rich elements of 3′-UTR [53]. Collectively, the SLC stimulation in CPEB2KO MCF10A cells could be due to an upregulation of COX2/EP4, and Wnt pathway genes.

Finally an examination of breast cancer and non-tumor breast tissues revealed a lower expression CPEB2 isoform A and higher expression of isoform B in cancerous tissues. This difference was more evident by measuring the ratios of A: B isoforms, presented in Fig. 8a. These results are in concordance with reported tumor-suppressor vs tumor-promoter functions of A and B isoforms [30]. However our probes could not demonstrate a preferential expression of A (A/E) or B (B/D) isoform in any of the tumor subsets based on the information on their ER, PR or HER2 status. Since these samples contained variable proportion of stromal and immune cells, it is possible that they may have masked the differences.

In summary, we demonstrate here that CPEB2, presumably the isoform A, plays a significant role in suppressing tumorigenesis in mammary epithelial cells by repressing EMT, migration, invasion, proliferation and SLC phenotype, possibly through multiple targets, one of them identified here as p53. Figure 8c presents a schema for the proposed mechanisms in tumor suppressor functions of CPEBE2.

## Conclusions

Present study, utilizing in vitro and in vivo functional assays in *CPEB2*-depleted mammary epithelial cells as well as human breast-derived non-tumor and cancerous tissues, demonstrates that CPEB2, presumably the isoform A, plays a key role in suppressing tumorigenesis in mammary epithelial cells. The underlying mechanisms involved suppression of EMT, migration, invasion, proliferation and SLC phenotype, via a newly-identified translational target p53.

## Additional files

Additional file 1: **Figure S1**. EMT marker proteins identified in MOCK and CPEB2KD MCF7 cells. Top panel: (**A)** and **(B).** Immunofluorescence Images for E-Cadherin and Twist 1 (stained green), nuclei stained blue with DAPI. CPEBKD cells show decreased E-Cadherin on cell membranes, and increased Twist 1 in cytoplasm. Bottom panel: Left, (**C)** and **(D):** Quantification of fluorescence (normalized to 1 for control mock cells showing significant reduction of E-Cadherin and increase in Twist 1 in KD cells (*p* < 0.05). Right, **(E)** Western blots showing decreased E-Cadherin and increased Twist 1, and a very minor increase (not significant) in Vimentin. (JPG 173 kb)

Additional file 2: **Figure S2**. Migration, invasion and proliferation in MCF7 cells after CPEB2KD. **(A)** Migration and **(B)** invasion measured in transwells respectively at 24 and 48 h reveal significant increases in CPEB2KD cells (p < 0.05). **(C)** Proliferation measured with 24 h BrdU uptake showed a minor increase, not significant (*p* = 0.06). Data represent means of 3 replicates (±SEM). (JPG 78 kb)

Additional file 3: **Figure S3**. VEGF-D, COX-2 (*PTGS2*) and EP4 (*PTGER4*) expression in MCF10A cells. Expression of mRNA (qRT-PCR; Mean ± SEM) for VEGF, COX-2 and EP4 in WT and CPEB2KO cells (*n* = 3). No change was seen in the expression of *VEGFB* or *VEGF-C,* however a 4.68-fold upregulation of *VEGF-D* was seen in the CPEB2KO cells compared to WT cells (*p* = 0.0020). *COX-2* mRNA expression increased 4.31-fold (*p* = 0.0024) and EP4 mRNA expression increased 3.45-fold (*p* = 0.011) compared to WT cells. (*) indicates *p* < 0.05. (**) indicates *p* < 0.01. (JPG 58 kb)

Additional file 4: **Table S1**. Differential gene and microRNA microarrays conducted with MCF7 and MCF7-COX-2 cells identified two COX-2 upregulated miRNAs miR526b and miR655. They collectively target 13 COX-2 downregulated genes, of which *CPEB2* appeared as the single common target. (DOCX 17 kb)

## Data Availability

All data are included in this manuscript including Additional files.

## Abbreviations

ALDH: Aldehyde Dehydrogenase
ANOVA: Analysis of Variance
ATCC: American Type Culture Collection
CCND1: Cyclin D1
CDH1: E-cadherin
CDH2: N-cadherin
CDK4/6: Cyclin Dependent Kinase 4/6
COX: Cyclo-oxygenase
CPE: Cytoplasmic Polyadenylation Element
CPEB: Cytoplasmic Polyadenylation Element Binding Protein
CPEB2KD: CPEB-2 Knockdown
CPEB2KO: CPEB-2 Knockout
CRISPR: Clustered Regularly Interspaced Short Palindromic Repeats
Ct: Threshold Cycle
DAPI: 4′,6-Diamidino-2-Phenylindole
DMEM: Dulbecco’s Modified Eagle Medium
EdU: 5′-ethynyl-2′-deoxyuridine
eEF2: Eukaryotic Elongation Factor 2
ELISA: Enzyme-Linked Immunosorbent Assay
EMT: Epithelial-to-Mesenchymal Transition
EP: Prostaglandin E Receptor
GAPDH: Glyceraldehyde 3-Phosphate Dehydrogenase
H&E: Hematoxylin & Eosin
HER2: Human Epidermal growth factor Receptor 2
HIF-1α: Hypoxia Inducible Factor 1α
HLA: Human Leukocyte Antigen
HS: Horse Serum
IL2Rϒ: Interleukin 2 Receptor ϒ chain
MCF: Michigan Cancer Foundation
MMP: Matrix Metalloproteinase
NOD: Non-Obese Diabetic
PG: Prostaglandin
qRT-PCR: Quantitative Reverse Transcription Polymerase Chain Reaction
SEM: Standard Error of the Mean
siRNA: Small Interfering RNA
SLC: Stem-Like Cells
TNBC: Triple Negative Breast Cancer
UTR: Untranslated Region
VEGF: Vascular Endothelial Growth Factor
ZEB1/2: Zinc-finger E-Box-Binding
