# Supplementary figures and images for: Tumor suppressor role of cytoplasmic polyadenylation element binding protein 2 (CPEB2) in human mammary epithelial cells
Source: BMC Cancer. 2019 Jun 11;19:561. doi: 10.1186/s12885-019-5771-5 (PMC6558855; doi:10.1186/s12885-019-5771-5)

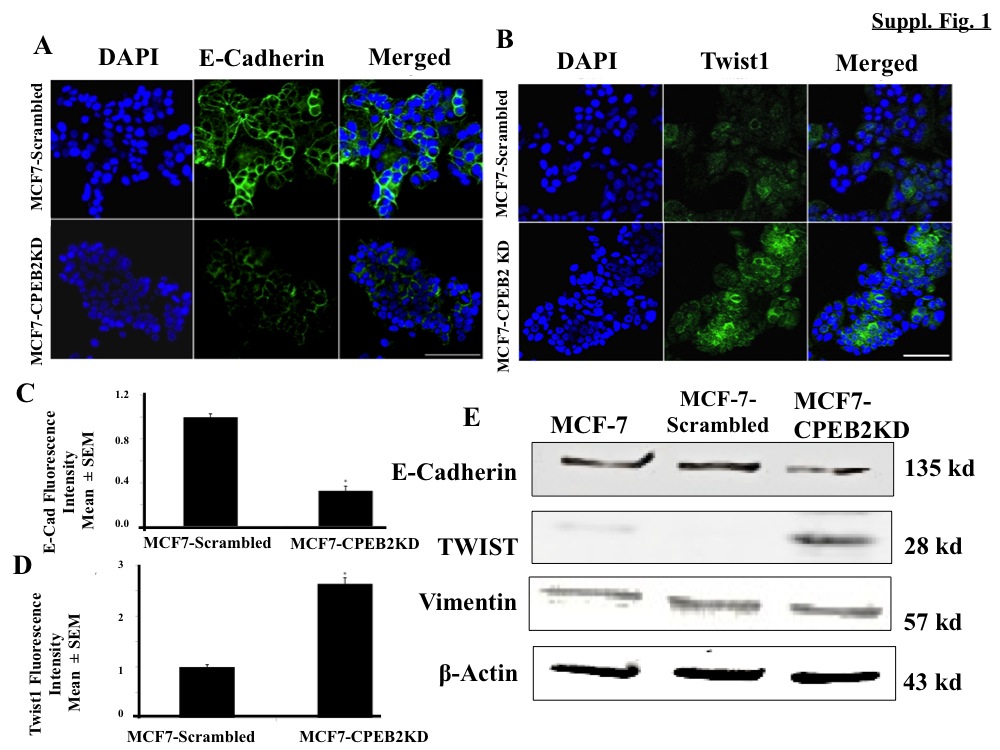

Supplement: Supplementary file 1 — Figure S1. EMT marker proteins identified in MOCK and CPEB2KD MCF7 cells. Top panel: (A) and (B). Immunofluorescence Images for E-Cadherin and Twist 1 (stained green), nuclei stained blue with DAPI. CPEBKD cells show decreased E-Cadherin on cell membranes, and increased Twist 1 in cytoplasm. Bottom panel: Left, (C) and (D): Quantification of fluorescence (normalized to 1 for control mock cells showing significant reduction of E-Cadherin and increase in Twist 1 in KD cells (p < 0.05). Right, (E) Western blots showing decreased E-Cadherin and increased Twist 1, and a very minor increase (not significant) in Vimentin. (JPG 173 kb) [file 12885_2019_5771_MOESM1_ESM.jpg]

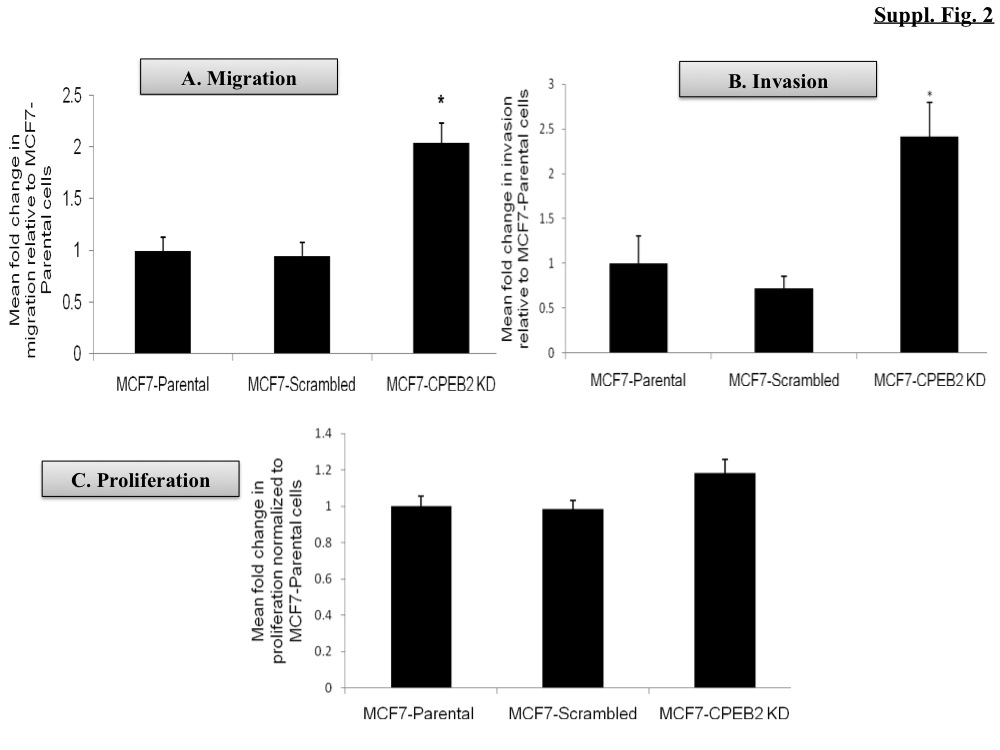

Supplement: Supplementary file 2 — Figure S2. Migration, invasion and proliferation in MCF7 cells after CPEB2KD. (A) Migration and (B) invasion measured in transwells respectively at 24 and 48 h reveal significant increases in CPEB2KD cells (p < 0.05). (C) Proliferation measured with 24 h BrdU uptake showed a minor increase, not significant (p = 0.06). Data represent means of 3 replicates (±SEM). (JPG 78 kb) [file 12885_2019_5771_MOESM2_ESM.jpg]

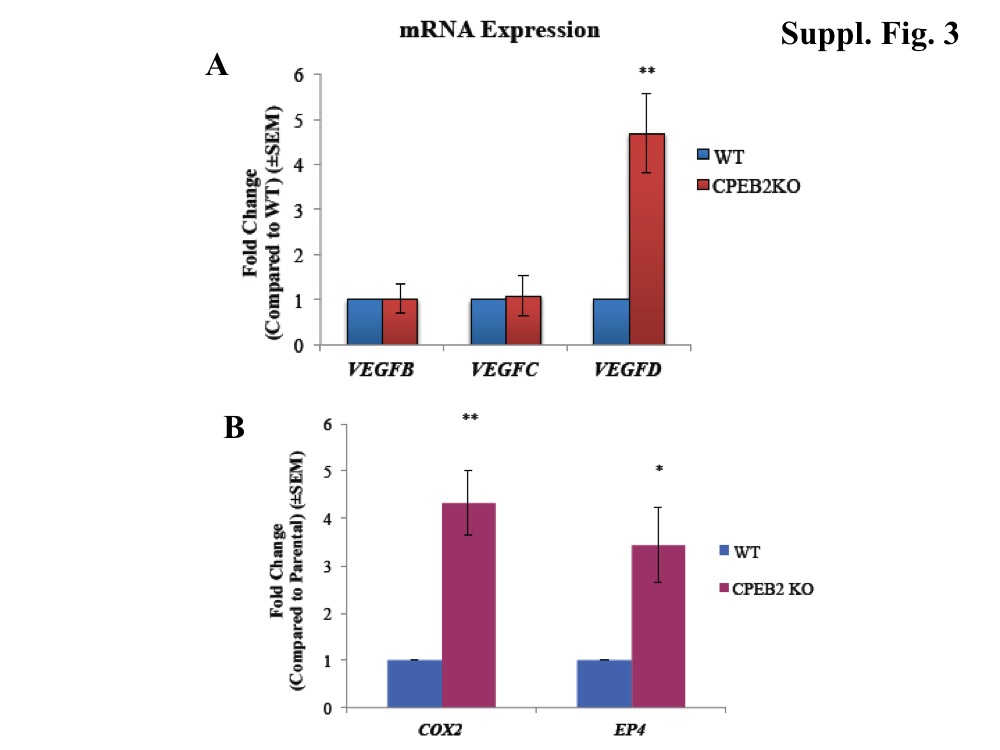

Supplement: Supplementary file 3 — Figure S3. VEGF-D, COX-2 (PTGS2) and EP4 (PTGER4) expression in MCF10A cells. Expression of mRNA (qRT-PCR; Mean ± SEM) for VEGF, COX-2 and EP4 in WT and CPEB2KO cells (n = 3). No change was seen in the expression of VEGFB or VEGF-C, however a 4.68-fold upregulation of VEGF-D was seen in the CPEB2KO cells compared to WT cells (p = 0.0020). COX-2 mRNA expression increased 4.31-fold (p = 0.0024) and EP4 mRNA expression increased 3.45-fold (p = 0.011) compared to WT cells. (*) indicates p < 0.05. (**) indicates p < 0.01. (JPG 58 kb) [file 12885_2019_5771_MOESM3_ESM.jpg]
