# Supplementary material for: Tumor suppressor role of cytoplasmic polyadenylation element binding protein 2 (CPEB2) in human mammary epithelial cells
Source: BMC Cancer. 2019 Jun 11;19:561. doi: 10.1186/s12885-019-5771-5 (PMC6558855; doi:10.1186/s12885-019-5771-5)
Supplement: Supplementary file 4 — Table S1. Differential gene and microRNA microarrays conducted with MCF7 and MCF7-COX-2 cells identified two COX-2 upregulated miRNAs miR526b and miR655. They collectively target 13 COX-2 downregulated genes, of which CPEB2 appeared as the single common target. (DOCX 17 kb) [file 12885_2019_5771_MOESM4_ESM.docx]

| **miRNAs up regulated in MCF7-COX2 cell line** | **Target Genes significantly downregulated in MCF7-COX2 cells** | |
| --- | --- | --- |
| hsa-miR-655 | Targeted by hsa-miR-655 | RILPL2 (Rab interacting lysosomal protein-like 2). Role in cellular protein transport. |
|  |  | EPB41L2 (Erythrocyte membrane protein band 4.1-like 2). Role in receptor/cytoskeleton interactions. |
|  |  | OPTN (optineurin). Roles in apoptosis, inflammation and vasoconstriction; in normotensive glaucoma. |
|  |  | TP53INP1 (Tumor protein p53 inducible nuclear protein). Role in p53 mediated tumor suppression |
|  |  | IFIT2 (Interferon-induced protein with tetratricopeptide repeats 2). Role in multiple tumor suppressor functions |
|  |  | DNAJB9 (DnaJ heat shock protein family (Hsp40) member B9). Role in preventing stress-induced cellular apoptosis |
|  |  | FGD6 (FYVE, RhoGEF and PH domain containing 6). Role in vacuolar protein sorting and endosome function. |
|  |  | WIPF1 (WAS/WASL interacting protein family member 1). Role in organization of the actin cytoskeleton. |
|  |  | NDRG1 (N-myc downstream regulated 1). Role in DNA repair. |
| hsa-miR-526b | Targeted by hsa-miR-526b | GCLM (Glutamate-cysteine ligase modifier subunit). Rate limiting enzyme of glutathione synthesis, a master ant-oxidant pathway. |
|  |  | CDK6 (Cyclin-dependent kinase 6). Role in cell cycle progression. |
|  | Targeted by both miRNAs | **CPEB2 (Cytoplasmic polyadenylation element-binding protein 2)** |

**Supplementary table 1. COX-2 downregulated gene targets of COX-2 upregulated microRNAs**
